# Can Cognitive Flexibility and Clinical Perfectionism Be Used to Identify People with Anorexia Nervosa?

**DOI:** 10.3390/jcm11071954

**Published:** 2022-03-31

**Authors:** Stephanie Miles, Maja Nedeljkovic, Andrea Phillipou

**Affiliations:** 1 Centre for Mental Health, Swinburne University of Technology, Hawthorn, VIC 3122, Australia; mnedeljkovic@swin.edu.au (M.N.); andreaphillipou@swin.edu.au (A.P.); 2 Department of Mental Health, St Vincent’s Hospital, Fitzroy, VIC 3065, Australia; 3 Department of Psychiatry, The University of Melbourne, Parkville, VIC 3010, Australia; 4 Department of Mental Health, Austin Health, Heidelberg, VIC 3084, Australia

**Keywords:** cognitive flexibility, perfectionism, anorexia, eating disorders, self-report

## Abstract

Poor cognitive flexibility and perfectionism are common features in anorexia nervosa (AN). The current study aimed to investigate cognitive flexibility and clinical perfectionism as potential predictors of AN. Twenty women with a current diagnosis of AN (*M* age = 28.25, *SD* = 7.62) and 170 community participants with no lifetime history of an eating disorder (*M* age = 29.23, *SD* = 9.88) took part in an online cross-sectional study that included self-report questionnaires of cognitive flexibility and clinical perfectionism. It was found that compared to the community sample, women with AN self-reported significantly poorer cognitive flexibility and significantly greater clinical perfectionism. In a regression model, clinical perfectionism (but not self-reported cognitive flexibility) significantly predicted group membership. The specificity and sensitivity of the model were high. These preliminary findings indicate that clinical perfectionism may represent a key feature of AN and may accurately discriminate between participants with and without AN, though more research is required.

## 1. Introduction

Anorexia nervosa (AN), as defined by the Diagnostic and Statistical Manual of Mental Disorders, 5th edition (DSM-5), is a serious eating disorder (ED) characterised by significantly low body weight, an intense fear of gaining weight, and disturbances in how one’s body weight or shape are experienced [1]. In addition to extreme fears of weight gain and thoughts that are preoccupied by food, weight, and related content [1], AN is characterised by various psychological and cognitive symptoms including poor cognitive flexibility and high levels of perfectionism [2,3,4,5,6,7,8,9]. Cognitive flexibility refers to the ability to effectively adapt to changes in the environment and/or changing task demands [10], whereas clinical perfectionism refers to a dysfunctional system of self-evaluation in which someone judges their self-worth primarily on their capacity to successfully meet personally demanding goals in spite of any negative consequences [11,12].

Poor cognitive flexibility is often exemplified in AN by strict calorie counting, rules for mealtimes, and intense exercise routines [13,14]. Compared to healthy controls (HCs), people with AN self-report poorer cognitive flexibility [2,3,4,5]. Some research has also shown that people with AN perform poorly on some neurocognitive tasks that assess cognitive flexibility; however, findings have been inconsistent across studies [2,15,16,17,18]. In addition, people with AN typically score higher than HCs on assessments of perfectionism [6,7,8,9,19]. However, research has generally used multidimensional assessments of perfectionism that incorporate both adaptive and maladaptive aspects of perfectionism. Adaptive perfectionism may be of value in the workplace, where high attention to detail ensures that mistakes are noticed in the early stages. Conversely, maladaptive perfectionism may be observed when a student spends months studying for exams late at night and thus misses out on vital sleep. Adaptive or ‘healthy’ perfectionism may have minimal relevance to mental illnesses and psychiatric research [12]. Hence, further research that focuses on the maladaptive aspects of perfectionism (i.e., clinical perfectionism) in AN is warranted.

Several lines of research have suggested that poor cognitive flexibility and perfectionism may act as risk factors for the development of AN [7,20,21,22,23]. Research including the unaffected sisters of people with AN has demonstrated that siblings of people with AN tend to perform poorer than HCs on tasks requiring cognitive flexibility [22,24,25]. These findings have led some researchers to argue that poor cognitive flexibility is an endophenotype of AN and a possible risk factor for the illness [7,24]. Perfectionism is thought to be present before the onset of AN [21] and may predict the onset of AN symptoms [26]. However, further research is required to determine if perfectionism is a risk factor for AN or a symptom of the illness. Poor cognitive flexibility and perfectionism are both considered to be key symptoms of AN [22,24,27] that may contribute to the illness [28,29,30]. Participants with AN have been found to significantly differ from HCs in cognitive flexibility and perfectionism; however, it remains unclear if these factors can be used to distinguish between people who have AN and those with no lifetime diagnosis of an ED.

The aim of the current cross-sectional study was to investigate cognitive flexibility and clinical perfectionism as predictors of AN. It was hypothesised that compared to a community sample, people with AN would report significantly poorer cognitive flexibility and significantly greater clinical perfectionism. It was also hypothesised that self-reported cognitive flexibility and clinical perfectionism would discriminate participants with AN from the community sample with no lifetime diagnosis of an ED.

## 2. Materials and Methods

### 2.1. Participants

The inclusion criteria for all participants were female, over the age of 18 years old, no history of a head injury accompanied by a loss of consciousness or a neurological condition, and no personal or family history of psychotic disorders. Participants in the AN group were required to have a current diagnosis of AN as determined by their own psychologist or psychiatrist and a current body mass index (BMI, kg/m^2^) ≤ 18.5. Community participants were required to have no personal or family history of an ED and were excluded if they were currently taking psychiatric or weight loss medication. Participants who reported that they were recovered from any ED were excluded from both groups.

The current study analysed data from 190 participants who were part of a larger online study of EDs. The sample for the current study comprised 20 women with a current diagnosis of AN (*M* age = 28.25, *SD* = 7.62) and 170 women from the community who self-reported no lifetime history of an ED (*M* age = 29.23, *SD* = 9.88). There were no significant differences between the groups in age; however, participants with AN had a significantly lower BMI than community participants (see Table 1).

### 2.2. Procedure and Measures

The study was approved by a university human research ethics committee. Data collection started on 9 March 2020 and ran until 29 June 2021. Participants were recruited from an established participant registry, a university research experience program, social media posts, public advertisements, and advertisements with Australian ED organisations. Undergraduate psychology students from the research experience program received course credit for their participation. Participants who were not part of the research experience program were not reimbursed for their participation.

Participation in the online study was anonymous and voluntary, and there were no adverse consequences for participants if they withdrew partway through the study or declined to participate. After providing informed consent, participants answered demographic and medical history questions followed by a battery of assessments. The questionnaires relevant to the present research are described below.

Self-reported cognitive flexibility was assessed using the Cognitive Flexibility Inventory (CFI; [31]), and clinical perfectionism was assessed by the Clinical Perfectionism Questionnaire (CPQ; Fairburn et al., unpublished, cited in [32]). In the instructions for the CPQ, participants were asked to disregard standards for eating, weight, or appearance in their self-assessment. Thus, the CPQ provided an assessment of clinical perfectionism which was independent of self-expectations and standards related to ED thoughts and behaviours. ED symptomatology and current height and weight (used to calculate current BMI) were assessed using the Eating Disorders Examination-Questionnaire (EDE-Q; [33]). Higher scores on the EDE-Q and CPQ represent greater severity/frequency of symptoms, and lower scores on the CFI indicate poorer cognitive flexibility. These scales are reliable and valid [31,34,35,36,37,38], and all scales and subscales had excellent internal consistency in the current study (Cronbach’s alphas > 0.80).

### 2.3. Statistical Analysis

Analyses were completed using SPSS version 27, and the alpha level was set at 0.05 for all analyses. Across the CFI and CPQ, the data were determined to be normally distributed and there were no univariate outliers (cases where z-score was greater than +/−3.29, *p* < 0.001; [39]) within the participant groups. To compare the AN group to the community group, one-way between groups analyses of variance (ANOVAs) were conducted. Where the assumption of homogeneity of variances was violated, Welch’s *F* is reported. Eta-squared effect sizes are reported and were defined as 0.01 = small, 0.06 = medium, and 0.14 = large [40].

A binary logistic regression comparing AN and community participants using CFI and CPQ together was conducted. Given the unequal group sizes, the prevalence rate of AN in the sample (0.10) was used as a cut-off point for the case classification. The predicted probability of AN diagnosis, or not, was computed for each participant. A receiver-operating-characteristics (ROC) was obtained, and sensitivity and one-specificity were calculated. The total area under the curve ROC is a measure of the overall performance of the model. A value of 0.5 under the ROC curve implies that the model performs no better than chance while a value of 1.0 indicates a perfect identification [41].

## 3. Results

### 3.1. Group Comparisons

There were significant differences between the groups in CFI (*F*(1, 188) = 4.06, *p* = 0.045, η^2^ = 0.02), and CPQ (*F*(1, 188) = 30.45, *p* < 0.001, η^2^ = 0.14) (see Table 1). Participants in the AN group scored significantly lower than the community group in CFI and significantly higher in CPQ, with small and large effect sizes, respectively.

### 3.2. Regression Models

The regression model (see Table 2) tested if CFI and CPQ could together predict AN. This model was statistically significant (χ^2^(2) = 27.58, *p* < 0.001); however, only CPQ was able to significantly predict the participant group’s status with an odds ratio of 1.21. CFI did not significantly predict the participant group’s status with an odds ratio of 0.98. The model correctly classified 75.8% of the total sample, with 75% sensitivity and 75.9% specificity. A ROC curve analysis resulted in a value of 0.81 as the area under the curve (CI = 0.70–0.92), indicating that the model was excellent at separating AN from community participants.

## 4. Discussion

The overall aim of this study was to investigate if cognitive flexibility and clinical perfectionism could differentiate individuals with and without AN. The results support the first hypothesis and indicate that, compared to a community sample, people with AN report significantly poorer cognitive flexibility. This finding is in line with past research that has found that people with AN self-report significantly poorer cognitive flexibility than healthy controls [3,4,5,42]. Although the current study supported a significant difference between the groups in self-reported cognitive flexibility, the effect size was small. Hence, the difference between the groups in self-reported cognitive flexibility may not be clinically meaningful.

Compared to the community sample, participants with AN reported significantly greater clinical perfectionism, with a large effect size. This finding is consistent with past research that has demonstrated that people with AN experience significantly higher levels of perfectionism than HCs [6,7,8,9,19]. Past research has investigated perfectionism from a multidimensional conceptualisation that includes both the adaptive and maladaptive aspects of perfectionism. Hence, the current study sought to focus on clinical perfectionism: a dysfunctional and maladaptive form of perfectionism. The results of this study build upon past work on perfectionism and demonstrate that people with AN judge their self-worth on their ability to work towards and meet personally demanding goals even in the face of negative consequences.

The results of the regression model partially support the second hypothesis that cognitive flexibility and clinical perfectionism would discriminate between women who did or did not have a diagnosis of AN. It was found that only clinical perfectionism significantly discriminated participants with AN from community participants. The specificity (the correct identification of community participants) and the sensitivity (the correct identification of participants with AN) of the model were high (75.9% and 75%, respectively), and the model was determined to be excellent at discriminating participants with AN from community participants. Hence, it is concluded that clinical perfectionism accurately discriminated between individuals with and without AN. Some research has shown that baseline maladaptive perfectionism predicts ED symptoms in the follow-up assessment [43,44]; however, to our knowledge, no research has investigated if cognitive flexibility or clinical perfectionism can predict current AN. Future research on this topic may wish to extend the work of the current study and investigate if clinical perfectionism can be used to discriminate between participants with acute AN, participants who are weight-restored, and participants who are fully recovered from AN. There is some evidence to suggest that people who have fully recovered from an ED exhibit significantly lower levels of perfectionism compared to people who are acutely ill [45]; however, further work specific to AN is required. Perfectionism is a transdiagnostic factor that has been found in EDs, anxiety disorders, depression, and obsessive-compulsive personality disorder [46]. However, it is unclear if the severity of clinical perfectionism could be used to differentiate AN from other psychiatric illnesses. For instance, Pike et al. [20] found that perfectionism (together with parental demands) discriminated between people with AN and people with other psychiatric illnesses. It would be of value for future research to further investigate this question.

The current study had several strengths, including the large community sample size and the focus on maladaptive perfectionism. However, there were also some limitations. The size of the AN group was much smaller than the community sample, and participants were only eligible to take part in the study if their sex was female. The inclusion of only females in this study reduces the generalisability of the results. Information on AN subtype (i.e., restricting type or binge-eating/purging type) was not collected from participants. Hence it is unknown which AN subtype participants were diagnosed with, and it is unclear if within-group subtype differences influenced the results. Previous research [20] has reported lower perfectionism scores in participants with binge-eating/purging type AN compared to restricting type AN. Thus, future research should aim to separate AN groups according to subtype. Participants in the community sample self-reported no history of an ED; however, the accuracy of this report could not be validated. Future research may benefit from the inclusion of clinical assessments to confirm the presence or absence of an ED. In addition, this study used a self-report assessment of cognitive flexibility. It is well known that self-report and neurocognitive assessments of cognitive flexibility do not relate well [3,47,48,49]; hence, the findings of this study cannot be generalised beyond self-reported cognitive flexibility. Future research may wish to replicate the current study using a neurocognitive task of cognitive flexibility.

The findings of this study indicate that although self-reported cognitive flexibility is generally poorer in people with AN as compared to controls, it may not represent a key feature of the illness. The effect size of the group difference for self-reported cognitive flexibility was small, and if poor cognitive flexibility was a key feature of AN, we would expect to be able to distinguish groups based on this factor. In addition, the results suggest that clinical perfectionism scores are higher in people with AN and that people with AN may experience a dysfunctional form of perfectionism, which impacts their functioning and self-worth. Clinical perfectionism may be a core feature of the illness that can be used to differentiate people with and without AN.

## Figures and Tables

**Table 1:** Sample characteristics and group comparisons.

	Group		Test Statistics		
	AN	Community	*F*	*p*	η^2^
Age	28.25 (7.62) [20–49]	29.23 (9.88) [18–57]	0.18	0.669	-
BMI	16.53 (1.44) [12.55–18.47]	25.45 (5.94) [13.16–48.83]	118.05 ^^^	<0.001	0.19
Age of AN onset	15.20 (4.56) [8–27]	-	-	-	-
Illness duration	13.25 (8.92) [0.83–33]	-	-	-	-
EDE-Q					
Restraint	4.37 (1.64) [0.60–6]	2.05 (1.52) [0–6]	40.92	<0.001	0.18
Eating concern	3.36 (1.81) [0.60–6]	1.37 (1.37) [0–5.60]	35.02	<0.001	0.16
Weight concern	4.06 (1.88) [0.80–6]	2.92 (1.69) [0–6]	7.87	0.006	0.04
Shape concern	4.38 (1.66) [1.13–6]	3.23 (1.76) [0–6]	7.17	0.006	0.04
Global score	4.04 (1.64) [0.88–6]	2.39 (1.37) [0–5.65]	24.78	<0.001	0.12
CFI	95.25 (3.21) [61–119]	102.02 (14.20) [64–137]	4.06	0.045	0.02
CPQ	36.85 (1.55) [21–46]	28.92 (5.98) [13–46]	30.45	<0.001	0.14

Note: mean (*SD*) [range] reported; age, age of AN onset, and duration of illness are reported in years; AN = anorexia nervosa; BMI = body mass index, kg/m^2^; CFI = Cognitive Flexibility Inventory; CPQ = Clinical Perfectionism Questionnaire; EDE-Q = Eating Disorders Examination-Questionnaire; ^^^ Welch’s *F* is reported as the assumption of homogeneity of variances was violated.

**Table 2:** Model of CFI and CPQ predicting AN.

Predictor	B	SE	Wald	df	*p*	Odds Ratio	95% CI	
							Lower	Upper
CFI	−0.02	0.02	1.40	1	0.236	0.98	0.95	1.01
CPQ	0.19	0.04	19.44	1	>0.001	1.21	1.11	1.31

Note: CFI = Cognitive Flexibility Inventory; CPQ = Clinical Perfectionism Questionnaire.

## Data Availability

Research data from this study are not shared.
